# Shared Decision Making Enhances Pneumococcal Vaccination Rates in Adult Patients in Outpatient Care

**DOI:** 10.3390/ijerph17239146

**Published:** 2020-12-07

**Authors:** Flora Kuehne, Linda Sanftenberg, Tobias Dreischulte, Jochen Gensichen

**Affiliations:** Institute of General Practice and Family Medicine, Munich University Hospital, 80336 Munich, Germany; Flora.Kuehne@med.uni-muenchen.de (F.K.); Tobias.Dreischulte@med.uni-muenchen.de (T.D.); Jochen.Gensichen@med.uni-muenchen.de (J.G.)

**Keywords:** shared decision making, pneumococcal, vaccination

## Abstract

Insufficient vaccination rates against pneumococcal disease are a major problem in primary health care, especially in adult patients. Shared decision-making (SDM) may address major barriers to vaccination. The objective of this review was to assess the impact of SDM on pneumococcal vaccination rates in adult patients. We conducted a systematic literature search in MEDLINE, EMBASE, CENTRAL, PsycINFO, and ERIC. RCTs and cluster RCTs were included, if they aimed to enhance pneumococcal vaccination rates in adult patients and comprised a personal interaction between health care provider (HCP) and patient. Three further aspects of the SDM process (patient activation, bi-directional exchange of information and bi-directional deliberation) were assessed. A meta-analysis was conducted for the effects of interventions on vaccination rates. We identified eight studies meeting the inclusion criteria. The pooled effect size was OR (95% CI): 2.26 (1.60–3.18) comparing intervention and control groups. Our findings demonstrate the efficacy of interventions that enable a SDM process to enhance pneumococcal vaccination rates; although, the quality of evidence was low. In exploratory subgroup analyses, we concluded that an impersonal patient activation and an exchange of information facilitated by nurses are sufficient to increase vaccination rates against pneumococcal disease in adult patients. However, the deliberation of options between physicians and patients seemed to be more effective than deliberation of options between nurses and patients.

## 1. Introduction

Pneumococcal infections account for a considerable burden of disease and associated economic burden worldwide [1] For example, in the US pneumococcal infections are estimated to contribute to 25,400 deaths annually and account for $3.8 billion of direct treatment costs per year [2].

Although pneumococcal infections can affect people of all ages, adults aged 65 years and above, as well as children below the age of two are at increased risk. Additionally, patients with chronic conditions have a higher risk for severe infections that can come with increased complications, long-term health constraints, and mortality [3,4].

Pneumococcal infections can be effectively prevented through vaccination [3,5,6]. Internationally available vaccines today comprise polysaccharide (PPV) and conjugate vaccines (PCV) protecting against different serotypes of *Streptococcus pneumoniae* [3,7]. Despite the possibility to treat pneumococcal disease with antibiotics, prevention by vaccination can additionally establish herd immunity by lowering pneumococcal carriage rates in the population and can counteract antibiotic resistance [3]. The vaccination against *Streptococcus pneumoniae* is recommended for elderly patients and patients with chronic conditions in most health care systems, with different application schemes for these respective patient groups [8,9,10,11].

Despite safety, accessibility and affordability of vaccines, pneumococcal vaccination rates remain below national targets in several high-income countries (e.g., in Germany 12.7% of the chronically ill and 50.9% of the elderly [12,13,14]). Pneumococcal vaccination rates tend to be especially low in high-risk patients (<65 years with a chronic health condition) ranging between about 13% in Germany and 30% in the US [12,13,14].

Insufficient vaccination rates are often a result of missed opportunities for vaccination (MOV), that might be attributable to health care provider (HCP) related factors, demand-related factors, and factors due to health system constraints [15,16]. Reported barriers to pneumococcal vaccination include missing recommendations and insufficient knowledge of HCP [17,18], as well as patients’ lack of awareness, assumptions of unnecessity, and doubts about the pneumococcal vaccine and its efficacy and safety [19,20].

In the consultation, the recommendation to get vaccinated, and the communication of risks and benefits can be constrained by HCP’s knowledge and confidence, communication style or lack of time [21,22,23]. HCP are recognized the most trusted source of health information for patients [20]. By taking into consideration that most patients are vaccinated in outpatient care [24], HCP in this setting are in a crucial position to inform and educate patients about vaccinations and to address patients’ doubts and concerns.

A frequently recommended approach of patient communication is described as shared decision making (SDM), which describes the involvement of the patient in the whole process of decision making, in which HCP and the patient take health care decisions based on partnership. The SDM approach emphasizes patients’ rights and autonomy and is considered as a strategy to reduce practice variations and promote evidence based medicine [25].

The concept of SDM was first developed by Charles et al. in 1997 [26] and further specified in the following years [25,27,28]. It is widely described as including the following three key aspects: information, deliberation, and taking a decision [29]. Therefore, the objective of our systematic review is to examine the effect of shared decision making processes in interventions to enhance pneumococcal vaccination rates in adults in outpatient care.

## 2. Materials and Methods

We conducted a systematic literature search in MEDLINE and EMBASE, the Cochrane Library, PsycINFO, and ERIC. Grey literature was identified from individual clinical trial registers through searches in clinicaltrials.gov, ICTRP (International Clinical Trials Registry Platform, WHO), the WHO NITAG resource center (https://www.nitag-resource.org/), CENTRAL, and PROSPERO. Additionally, we screened the references of relevant systematic reviews and studies to identify further potentially eligible studies.

Studies that aimed to enhance pneumococcal vaccination rates in adult patients (18 years and above) in outpatient care of high-income countries (according to the World Bank classification [30]) were included. Studies focusing on children (or their parents), pregnant women, medical students or health care workers, cognitively impaired patients, and drug users were excluded, as we supposed a considerable heterogeneity of patient groups concerning (national) vaccine recommendations, personal interests and believes about vaccination. Apart from that, interventions conducted in hospitals, nursing homes, workplaces, or homeless shelters were excluded to keep the clinical setting as homogenous as possible and exclude potential influence of institutional policies on vaccination requirements.

We only included randomized controlled trials (RCTs) and cluster RCTs. Control groups could receive usual care, an active control intervention, or an alternative intervention. Inclusion of eligible studies was not restricted concerning the year of publication.

A broad definition of SDM was used in the present work to identify studies with interventions aligning to that approach. As such, interventions had to feature personal interaction between patient and HCP aiming at an active participation in the decision making process.

To assess the SDM process in interventions, we used an adapted, unvalidated scale (Supplement S1, developed previously by Martinez-Gonzalez et al. [31] for the purpose of categorizing interventions according to key criteria of SDM based on their description.

As we considered the aspect of “taking a decision” at least reflected in the resulting vaccination rates, the present work focused on the decision process in interventions [32]. In many definitions of SDM there are aspects even before “information” and “deliberation”, representing some kind of patient activation (e.g., “two participants are involved” [26], “announcement, that there is a decision to be made” [25,33] or “encouragement to talk” [31]). Consequently, in this review three aspects characterize a SDM process that must be included in the interventions for studies to be eligible.

1. Patient activation.
2. Bi-directional exchange of information.
3. Bi-directional deliberation of options.

For this assessment, the intervention’s description, its content and mode of delivery were examined. It was conducted by two reviewers (FK, LS) independently, and disagreements were resolved by discussion. Interventions with a component targeting HCP had to be directed at physician or non-physician HCP who are allowed to vaccinate, e.g., nurses or pharmacists in certain health care systems.

Retrieved records were organized in a reference management software (EndNote^TM^, Clarivate^TM^, London, UK) and duplicates removed. Two reviewers (FK, LS) independently screened titles and abstracts for inclusion and exclusion criteria. Inconsistencies were solved by discussion. We procured potentially relevant articles, which included contacting authors of ongoing studies and abstracts without published full text. To determine the final study selection, we conducted the assessment of inclusion and exclusion criteria independently (FK, LS) and resolved discrepancies by consensus discussion (FK, LS, and JG).

Pneumococcal vaccination rates had to be reported for all groups to be included. Effect sizes comprised odds ratio (OR) and relative risk (RR) in included studies.

Data was extracted by two review authors (FK, LS) independently using a custom-made Excel and Word (Microsoft, Redmont, WA, USA) extraction form based on reference [34]. To assess the risk of bias in included studies we used the Cochrane Risk of Bias Tool 2 [35]. We also considered specific biases relating to cluster RCTs.

We used the GRADE [36] approach to assess the quality of evidence for the primary outcome. The assessment was conducted by two authors (FK, LS) independently and disagreements resolved by discussion.

We initially conducted a narrative synthesis of the included studies and the characteristics of the interventions. A quantitative synthesis (meta-analysis) of the effects on pneumococcal vaccination rates was conducted using the inverse-variance method and a random effects model.

For RCTs, ORs were calculated by absolute, unadjusted data, whereas for cluster RCTs adjusted ORs and CIs were used (as reported in the respective papers) and SEs calculated by Review Manager 5.3 (The Cochrane Collaboration, Copenhagen, Denmark). For one cluster RCT, the adjusted OR was calculated by absolute numbers and the ICC, as reported, using the method for adjusting data of cluster RCTs recommended by the Cochrane Handbook [35]. If studies reported only the results of per protocol analyses, we calculated effect sizes using an intention-to-treat approach from absolute numbers (number of patients randomized per group, as reported in the respective papers). Data was presented in tables and forest plots where appropriate.

In subgroup analysis we compared interventions according to the HCP responsible for the deliberation (as the central aspect in the SDM process) as well as the activation and information aspect. As only one study featured HCPs in the pharmacy setting, pharmacists or pharmacist assistants were not considered in subgroup analysis. A sensitivity analysis was conducted to control for studies with a high risk of bias in more than two of the assessed domains.

We submitted our study protocol to PROSPERO in advance (registration number: CRD42020175555, submitted 20 March 2020), where a detailed description of methods is documented.

## 3. Results

**Figure 1:**
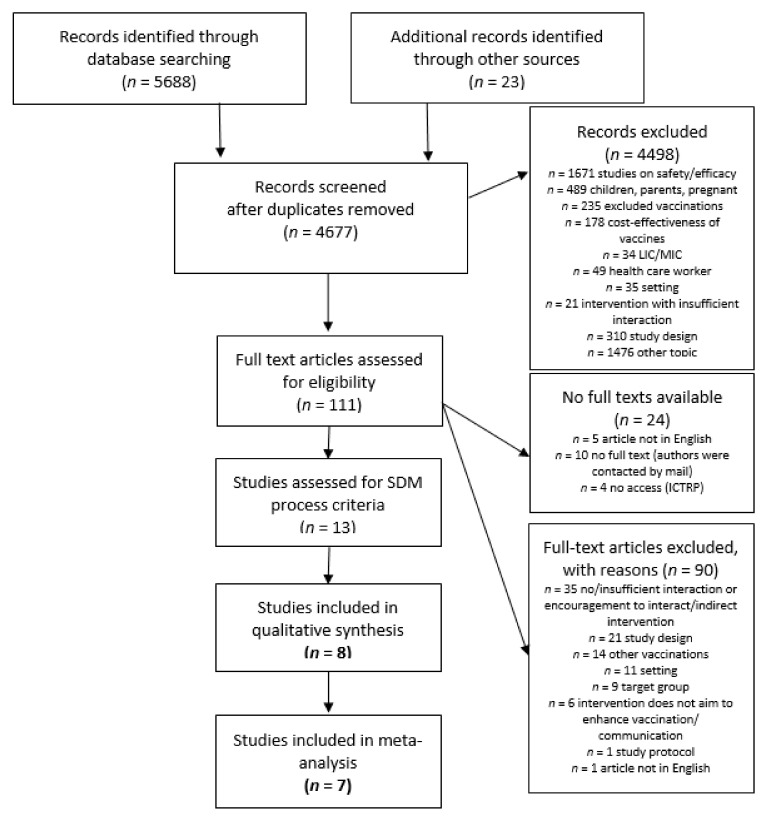
Flowchart.

Our literature search resulted in 5688 records, with 4677 remaining after removing duplicates. Titles and abstracts were screened for inclusion and exclusion criteria resulting in 135 articles for full text assessment. Of 111 available full texts, 13 studies matched the selection criteria. Eight studies were included in the final analysis, because they met our criteria for a SDM process. All studies were included in meta-analysis. The literature search and selection process is demonstrated in Figure 1.

### 3.1. Included Studies

Of the included studies, five were randomized at individual level (by patient/household [37,38,39,40,41]) and three at cluster level (by practice [42,43] or intervention week [43]). Studies were published between 1999 and 2018. Studies were conducted in general practitioner’s (GP) practices [38,41,43,44], specialist clinics [37,42], another ambulatory clinic [39] or a pharmacy [40]. They were located in Australia [43], Belgium [37], Germany [38], Hong Kong [44], Switzerland [41], and the United States [39,40,42].

Studies either targeted patients who were elderly and had chronic conditions [38,41,44], patients who were elderly or had a chronic condition [39], or patients with chronic conditions irrespective of age (asthma or COPD [40]; COPD [43]; inflammatory bowel disease [37]; or lymphoma remission [42]). Table 1 gives an overview about characteristics of included studies.

### 3.2. Characteristics of Interventions

Interventions comprised face-to-face sessions between HCP and patient to inform and deliberate on vaccinations [37,38,41,42,43,44], telephone interventions by HCP to provide information and decision support for the patient [40,41,43,44], or discussion empowering educational material for patients [38,39,41], which provided some information on vaccination and called the patient to raise questions or concerns in the consultation. Additionally, some interventions comprised trainings about vaccination and/or communication directed at physicians, pharmacists, or nurses. [38,41,42,43,44].

As per inclusion criteria, all interventions displayed indications for patient activation, bi-directional exchange of information and deliberation of options. To address these aspects, responsibilities were often shared between different HCP in a practice team [37,38,41,43] or facilitated by one HCP [39,40,42,44].

### 3.3. Effect of Interventions

Pneumococcal vaccination rates were significantly increased in the intervention group compared to the control group in five of the eight studies [37,38,39,41,44], increased without reaching statistical significance in two studies [40,43] and decreased non-significantly in one study [42]. The pooled effect size for all included studies was OR (95% CI): 2.26 (1.60–3.18).

Attained vaccination rates ranged from 3.8% in a control group [39] to 72.2% in an intervention group [43].

**Figure 2:**
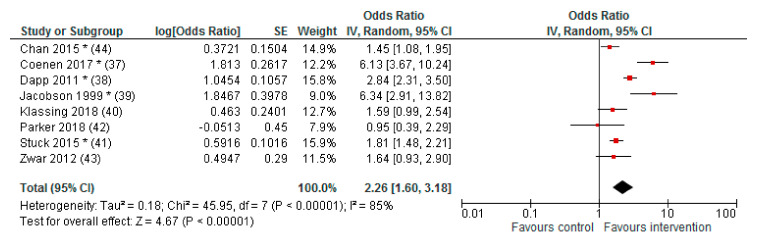
Forest plot (meta-analysis) of effects on vaccination rates.

The results of the meta-analysis are demonstrated in Figure 2.

Calculated by Review Manager 5.3 (The Cochrane Collaboration, Copenhagen, Denmark) inverse-variance method (IV), random effects model; * *p* < 0.05; Cluster RCT: adjusted ORs as reported, SEs calculated [42,43], adjusted OR calculated by absolute numbers, and ICC [44]; RCT: ORs calculated by absolute numbers (no adjustments).

### 3.4. Subgroup Analyses

The results of the subgroup analyses are shown in Table 2.

Interventions where patients were activated by nurses or an impersonal activation method (e.g., short message reminder) both showed significant effects on pneumococcal vaccination rates. Results suggest an even higher increase of vaccination rates through interventions where patient activation was conducted impersonally. Patient activation by physicians did not demonstrate a significant effect in the included studies.

Studies with bi-directional exchange of information facilitated by nurses in interventions showed significantly increased vaccination rates in intervention groups compared to control groups. Interventions with physicians responsible for that aspect of a SDM process did not show statistically significant effect sizes.

Concerning the deliberation of options (the central aspect of a SDM process), we identified a significant effect on vaccination rates of interventions, where deliberation was facilitated by physicians. The meta-analyzed effect size of interventions where the deliberation of options was facilitated by nurses did not reach statistical significance.

### 3.5. Sensitivity Analysis

When studies with a high risk of bias in more than two of the assessed domains were excluded from quantitative analysis, the pooled OR was 1.86 (95% CI: 1.40–2.48) for intervention compared to control (Appendix A). For the subgroup comparison of different HCP facilitating the deliberation within the SDM process, we found a remaining higher effect for interventions with deliberating physicians (OR (95% CI): 2.26 (1.45–3.53)) compared to deliberating nurses (OR (95% CI): 1.49 (1.15–1.93)), with both effects reaching statistical significance (Appendix A).

### 3.6. Risk of Bias in Included Studies

Randomization that was not computer or program generated (alternating [39]; assignment by blinded physician [37]) or showed major baseline differences [43] were attributed a high risk of bias concerning this domain. Deviations of intended interventions resulted due to patients changing groups [37]; interventions affecting HCP who treat both groups [39,41]; patients in the intervention group not receiving the intervention [44]; and missing implementation of the intervention due to physicians’ perceived incompatibility to recent or current therapies (Rituximab) [42].

Studies with more than 20% of missing outcome data were attributed a high risk of bias concerning this domain. A high risk of bias regarding the “measurement of outcome” was attributable to patient reported outcome measurement. Because all studies reported achieved pneumococcal vaccination rates, risk of reporting bias was assessed as low. Other bias comprised an untransparent selection of study participants [37] or unclear components in the reporting of the studies [40,42].

Using the GRADE approach, overall quality of evidence was assessed as low for included studies.

**Figure 3:**
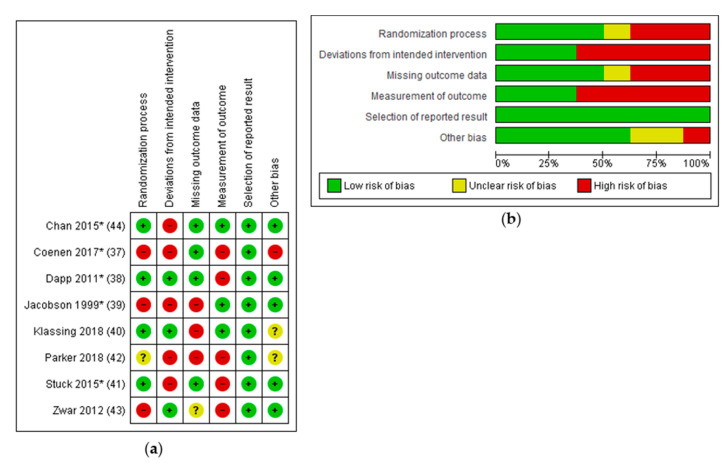
Risk of Bias assessment; (**a**) Risk of Bias summary; (**b**) Risk of Bias graph.

Figure 3 gives an overview of the Risk of Bias assessment.

## 4. Discussion

### 4.1. Summary of Results

We found that interventions enabling a SDM process increase pneumococcal vaccination rates compared to control groups (OR (95% CI): 2.26 (1.60–3.18). Interventions comprised (combinations of) several components including face-to-face sessions, discussion empowering educational material or telephone outreach for patients, as well as trainings about vaccination and communications skills for HCP.

The effect of the SDM elements depended on the type of implementation and the profession of the facilitating HCP. Effective strategies in interventions to increase vaccination rates seem to be an impersonal patient activation method, an exchange of information facilitated by a non-physician HCP and a deliberation of options enabled by a physician.

### 4.2. Interpretation of Results

Overall, the analyzed studies showed enhanced pneumococcal vaccination rates in the intervention group, although the quality of evidence was low. Two studies showed non-significant effects, of which one attributable to a major risk of bias [42]. As in this study physicians hesitated to recommend the pneumococcal vaccine within the follow up time due to recent or current therapy (Rituximab) of their patients and the effectiveness of the intervention on pneumococcal vaccination rates may be suspected within a prolonged follow up time. In other included studies, a less pronounced risk of bias was notable, [37,39]. A sensitivity analysis controlling for studies with bias concerns still showed a significant effect on pneumococcal vaccination rates.

When interpreting studies that implemented trainings for HCP, further aspects have to be considered. As such, only part of these studies was implemented within a cluster randomized study design (by HCP or practice). This possibly led to a dilution of the intervention effect, because the control group was also treated by trained HCP [38,41,44].

Furthermore, half of the studies did not only focus on vaccination, but implemented multicomponent interventions for several health issues (e.g., COPD management including smoking cessation and optimizing medication [43], cancer screening, cardiovascular health prevention and health maintenance [38,41], or exercise, nutrition, smoking cessation, and lymphoma screening [42]).

To enhance practicability in clinical practice and to reduce time resources of physicians, non-physician HCP are increasingly involved in reminding/recalling patients, patient education, and administration of vaccines [25,45,46,47]. Likewise, in our reviewed studies, components of interventions and aspects of SDM processes were often addressed by different HCP. Especially in patient education, the check up on understanding, encouragement to address concerns and support to form preferences the value of the nurses’ role has been highlighted [25,48,49,50,51,52]. Likewise, we found particularly high effects on vaccination rates of interventions with nurses enabling the bi-directional exchange of information within the SDM process.

The deliberation of options is recognized a central aspect in the SDM process [31] and the latest point were indications and contraindications in a decision about vaccinations have to be considered. Among HCP, physicians are trusted highly, as they are supposed to have more medical knowledge [21,53,54,55]. In addition, HCP with more knowledge on vaccination were found to recommend vaccinations more frequently and also feel confident to manage difficult conversations with patients [56]. This could explain our findings suggesting higher effects on vaccination rates of interventions, where the deliberation was facilitated by a physician.

### 4.3. Comparison to Previous Research

To our knowledge, this is the first systematic review assessing the impact of SDM interventions on pneumococcal vaccination rates. Other systematic reviews examining interventions to enhance vaccination rates reported comparable results for dialogue-based interventions. Lau et al. [46] found a significant increase in pneumococcal vaccination rates through patient outreach (OR (95% CI): 1.80 (1.54–2.11)), clinician training (OR (95% CI): 1.54 (1.19–1.99)), or team change (e.g., task shifting or increased responsibilities for non-physician HCP)(OR (95% CI): 2.09 (1.48–2.95). Patient outreach methods showed higher effect sizes, when personal contact was involved (e.g., face-to-face session, telephone outreach). A Cochrane Review by Jacobson et al. [45] on patient reminder and recall interventions included only two studies examining pneumococcal vaccinations in adult patients. In this work an intervention implementing a telephone intervention lead to an effect of OR (95% CI): 2.3 (2.0–2.7) on vaccination rates [57] and patient reminder letters combined with a training for HCP lead to a mean difference (MD) of 20% between groups [58].

### 4.4. Strenghts and Limitations

The strengths of this systematic review comprise its broad search strategy and the way we operationalized a SDM process. As a result, we were able to review the evidence for interventions aligning to a SDM approach, although not primarily intended as SDM interventions by researchers. However, we have to address some limitations of our review. First, we noticed a considerable heterogeneity between studies. Heterogeneity originated in patient groups, interventions, settings, and study designs and was also indicated by a notable I^2^ in the meta-analysis. A wide range of attained vaccination rates additionally suggest diverse baseline vaccination rates in included studies. Heterogeneity must also be considered in terms of control conditions, which could influence resulting effect sizes. Intervention groups were compared to groups receiving usual care, an alternative intervention, or an active control intervention. Even in studies, providing usual care in the control arm, notable differences might exist depending on the setting, HCP, and health care system, as there is no common definition for “usual care”.

Heterogeneity concerning baseline variables of study populations exist and have not been controlled for, as we presented unadjusted ORs calculated from absolute numbers to enhance comparability of effect sizes of RCTs. Thus, for cluster RCTs we used adjusted ORs and CIs, that were adjusted for clustering effect [42,44] and additionally for baseline variables [43].

Another limitation is, that we could not use any validated measurement method to assess the SDM process, but had to rely on the interventions’ description, its content and mode of delivery. Thereby, the actual situation of patient-HCP interaction, as well as perspectives of the involved partners in the SDM process might not be reflected. Although specific tools and measurement methods exist [32,59,60], none of the included studies used any to objectify SDM.

Overall, the total number of included studies in this review was small, with only eight identified studies meeting the inclusion criteria. Therefore, results must be interpreted carefully; especially those of the subgroup analyses, as very few studies are contained in most comparisons and differences between subgroups could originate from other characteristics of interventions.

As we only included studies to publications written in English or German, we may have missed relevant publications. However, studies from several non-English speaking countries were included in our analysis. A funnel plot of study results did not indicate publication bias, showing evenly distributed effect sizes (Appendix B).

### 4.5. Further Research

Further studies are needed to substantiate our findings and examine the effectiveness of certain components of interventions. Future clinical trials should apply standardized measurement tools for SDM, ideally using a dyadic approach (patient’s and HCP’s perspective) and examine SDM for other vaccinations and patients groups.

### 4.6. Implications for Policy and Practice

Amidst the COVID-19 pandemic awareness for vaccine-preventable diseases has risen and the demand for vaccines has increased substantially worldwide [61,62,63]. Especially among high-risk populations vaccination against pneumococcal and influenza has recently been promoted to prevent co-infections with COVID-19 and to save health care capacities within systems [64]. While we have shown similar effects of SDM interventions to increase influenza vaccination rates (Sanftenberg et al. 2020; unpublished data), favourable effects of SDM processes may be expected whenever patient awareness, knowledge, beliefs, and trust are barriers to the uptake of recommended vaccinations.

HCP could align their practice to a SDM approach by actively engaging their patients and empowering them to address their questions, preferences, and concerns regarding the pneumococcal vaccination.

SDM can be facilitated stepwise by a team of HCP, preparing the patient for the deliberation of options that might take place together with a physician.

(Continued) medical education for all HCP offering vaccination services is crucial to provide HCP with the knowledge and communication skills needed to confidently inform about risks and benefits of the vaccination and deliberate options [21,25]. Awareness should be risen for SDM in vaccination decisions, enabled by policies to invoice vaccination consultations separately from the application of the vaccine [65].

## 5. Conclusions

Pneumococcal vaccination rates can be effectively increased by elements of SDM. Responsibilities for key aspects of SDM can be shared in HCP teams, and thereby enable practicability in clinical practice. HCP knowledge and communication skills are key elements, as well as enabling factors like available time resources and payment schemes for consultations.

## Figures and Tables

**Table 1:** Characteristics of included studies.

Study	Country	Patients/Age	Intervention	Vaccination Rate (IG)	Vaccination Rate (CG)	*n* (IG)	*n* (CG)	Effect Size/*p*-Value for Vaccination Rate	Follow up
Chan 2015 * (44)	Hong Kong	65+ with chronic disease	Telephone outreach and face-to-face session	57.2%	48.1%	1251	1266	ARR (95% CI): 1.20 (1.06–1.37)	3 months
Coenen 2017 * (37)	Belgium	Inflammatory bowel disease patients	Face-to-face session	62%	23%	86 (PP) 104 (ITT)	107 (PP) 206 (ITT)	*p* < 0.001 (PP) OR (95% CI): 6.13 (3.67–10.24) (ITT)	8 months
Dapp 2011 * (38)	Germany	60+	Computer generated feedback for patient and HCP, group session or home visit for patient and training for HCP, discussion empowering educational material	47%	23.8%	568	1342	OR (95% CI): 2.8 (2.3–3.5), *p* < 0.001	1 year
Jacobson 1999 * (39)	USA	65+ or chronic disease	Discussion empowering educational material (to be used in consultation)	19.9%	3.8%	221	212	RR (95% CI): 5.28 (2.80–9.93), *p* < 0.001	1 day
Klassing 2018 (40)	USA	18+ with Asthma/COPD	Telephone outreach	59.7%	55.7%	77 (PP) 216 (ITT)	70 (PP) 269 (ITT)	*p* = 0.76 (PP) OR (95% CI): 1.59 (0.99–2.44)	5 months
Parker 2018 (42)	USA	18+ lymphoma survivors	New face-to-face consultation and communication skills training for HCP	14%	14%	117	81	logistic HLM: OR (95% CI): 0.95 (0.39–2.32) PH model, HR (95% CI): 0.91 (0.51–1.63)	12 months
Stuck 2015 * (41)	Switzerland	65+	Computer generated feedback for patient and HCP, telephone outreach and face-to-face session (home visit) for patients and training for HCP, discussion empowering educational material	31.3%	20.2%	827	1320	OR (95% CI): 1.90 (1.52–2.37), *p* < 0.001	2 years
Zwar 2012 (43)	Australia	40–80 years with COPD	Face-to-face session (home visit)	72.7%	61.7%	161	169	OR 1.64 (0.93–2.89), *p* = 0.09	12 months

* *p* < 0.05; ITT: intention-to-treat; PP: per-protocol; IG: intervention group; CG: control group.

**Table 2:** Subgroup analyses (meta-analysis).

Subgroup	Number of Studies	OR (95% CI)	I^2^
Activation			
impersonal	3 [38,39,41]	2.79 (1.73–4.50) *	88%
by nurse	2 [43,44]	1.49 (1.15–1.93) *	0%
by physician	2 [37,42]	2.50 (0.40–15.52)	92%
Information			
by nurse	5 [37,38,41,43,44]	2.32 (1.57–3.43) *	88%
by physician	2 [39,42]	2.48 (0.39–15.95)	90%
Deliberation			
by nurse	3 [37,43,44]	2.42 (0.99–5.89)	91%
by physician	4 [38,39,41,42]	2.38 (1.50–3.77) *	85%

Activation: patient activation; Information: bi-directional exchange of information; Deliberation: bi-directional deliberation of options; * *p* < 0.05; I^2^: measurement of heterogeneity.

**Figure A1:**
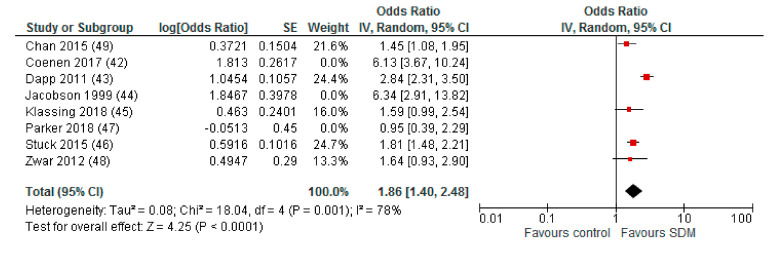

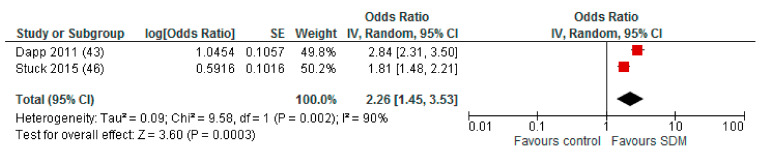
Subgroup: Deliberation by physicians.

**Figure A2:**
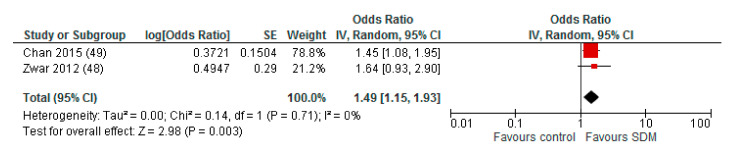
Subgroup: Deliberation by nurses.

## Supplementary Materials

The following are available online at https://www.mdpi.com/1660-4601/17/23/9146/s1, S1: SDM Assessment Tool, S2: Search strategy.
