# Supplementary material for: Shared Decision Making Enhances Pneumococcal Vaccination Rates in Adult Patients in Outpatient Care
Source: Int J Environ Res Public Health. 2020 Dec 7;17(23):9146. doi: 10.3390/ijerph17239146 (PMC7729624; doi:10.3390/ijerph17239146)
Supplement: Supplementary file 1 [file ijerph-17-09146-s001.pdf]

Supplement 1: SDM Assessment Tool

	**Patient activation**	**Bi-directional exchange of information**	**Bi-directional deliberation of options**
Full SDM Process	1	1	1
Unclear deliberation in SDM Process	1	1	?
Unclear information and deliberation in SDM Process	1	?	?
Unclear SDM Process	?	?	?
No SDM Process	0/?/1	0/?/1	0

Criteria met = 1, criteria not met = 0, unclear = ?

Similar to Martinez-Gonzalez, et al 2018 [1].

1. Martinez-Gonzalez NA, Plate A, Senn O, Markun S, Rosemann T, Neuner-Jehle S. Shared decision-making for prostate cancer screening and treatment: a systematic review of randomised controlled trials. Swiss medical weekly. 2018;148:w14584.

Supplement 2: Search strategy

Textword search strategy*:*

Columns are linked with AND, word in columns with OR.

Adult<br>*<br>elderl<br>*<br>aged	pneumococc<br>*<br>shingles<br>herpes zoster<br>influenza<br>flu<br>measles<br>MMR<br>pertussis<br>diphteria<br>tetanus<br>poliomyelitis<br>polio<br>rubella<br>mumps<br>whooping<br>cough	dialogu *<br>patient-provider<br>interpersonal<br>face-to-face<br>face to face<br>in person<br>participative<br>communication-<br>based<br>communication<br>based<br>dialogue-based<br>dialogue based<br>telephon *<br>personal *<br>individual *<br>counsel *<br>comunicati *<br>professional-patient<br>professional patient<br>interact*	decision support<br>joint decision<br>choice<br>choice behaviour<br>informed choice<br>individual * decision<br>provision of<br>information cognitive<br>empowerment<br>patient<br>empowerment<br>informati *<br>risk communication<br>decision* coaching<br>coaching<br>improve<br>comprehensi *<br>improve understand<br>*<br>improve knowledge<br>enhance comprehensi<br>*<br>enhance understand *<br>enhance knowledge<br>shared decision-<br>making<br>shared decision<br>making<br>shared<br>decisionmaking<br>patient participation<br>patient involvement<br>participat * decision<br>making<br>participat * decision-<br>making participat *<br>decisionmaking<br>informed consent<br>individual choice	immune *<br>vaccin *<br>unvaccin *<br>unimmune *	controlled trial<br>rct<br>controlled<br>clinical trial<br>randomized trial<br>randomized<br>controlled trial<br>groups<br>trial<br>double blind<br>random *

Textword search strategy used for:

- Medline
  Search 2: +filter for CCT, RCT, review, systematic review
- Embase
  Search 2: +filter for CCT, RCT, systematic review
- CENTRAL
- PsycINFO
- ERIC

Medline thesaurus search (2 February 2020) via ovid + Epub Ahead of Print, In-Process & Other Non-Indexed Citations, Daily and Versions

Columns are linked with AND, word in columns with OR.

exp Adult/ exp Young Adult/	exp Professional Patient Relationship/ exp Patient Participation/ exp Health Personnel Attitude/ exp Health Knowledge, Attitudes, Practice/ exp Patient Centered Care/ exp Persuasive Communication/ exp Interpersonal Communication/ exp Health Communication/	exp Informed Consent/ exp Decision Support System/ exp Diplomacy/ exp Decision Making/ exp Negotiating/ exp Patient Advocacy/ exp Consumer Health Information/ exp Health Literacy/ exp Medical Information/ exp Attitude to Health/ exp Information Seeking/ exp Patient Attitude/ exp Decision Support Technique/ exp Patient Education/	exp Immunization/ exp Vaccination/ exp Vaccination Coverage/ exp Vaccination Refusal/ exp Anti-Vaccination Movement/ exp Vaccine Hesitancy/	exp Randomized Controlled Trial/

Embase (2 February 2020) via ovid

exp Adult/ or exp aged/

exp Professional Patient Relationship/ or exp Patient participation/ or exp Health Personnel Attitude/ or exp Health Knowledge, Attitudes, Practice/ or exp Patient Centered Care/ or exp Persuasive Communication/ or exp Health Communication/

exp Informed Consent/ or exp Decision Support System/ or exp Decision Making/ or exp Negotiating/ or exp Patient advocacy/ or exp Health Education/ or exp Consumer Health Information/ or exp Health Literacy/ or exp Medical Information/ or exp Attitude To Health/ or exp Information Seeking/ or exp Patient Attitude/ or exp Decision Support Technique/ or exp Patient Education/

exp Immunization/ or exp Vaccination/ or exp Vaccination Coverage/ or exp Vaccination refusal/ or exp Anti-Vaccination Movement/ or exp Vaccine hesitancy/

exp Randomized Controlled Trial/

Search 2: + filter for RCT, controlled trial and systematic review

PsycINFO thesaurus search (2 February 2020) via EBSCO

DE "Immunization" AND (DE "Communication" OR DE "Interpersonal Communication" OR DE "Verbal Communication" OR DE "Persuasive Communication" OR DE "Interpersonal Influences" OR DE "Interpersonal communication" OR DE "Communication Skills" OR DE "Communication Skills Training" OR DE "Verbal Communication" OR DE "Electronic

Communication“ OR DE “Interpersonal Interaction“ OR DE “Conversation“) AND (DE “Individual Education Program“ OR DE “Individualized Instruction“ OR DE “Client Participation“ OR DE “Client Satisfaction“ OR DE “Client Education“ OR DE “Decision Making“ OR DE “Choice Behavior“ OR DE “Decision Theory“ OR DE “Informed Consent“ OR DE “Decision Support Systems“)

ERIC thesaurus search (2 February 2020) via EBSCO

(DE "Adults" OR DE "Adult Students" OR DE "Older Adults" OR DE "Young Adults")
AND (DE “Immunization Programs“)
AND (DE “Communication (Thought Transfer)“ OR DE “Interaction“ OR DE “Interpersonal Relationship“ OR DE “Patient Education“ OR DE “Counselling“ OR DE "Discussion" OR DE "Interpersonal Communication" OR DE "Verbal Communication" OR DE "Communication Skills" OR DE "Counselor Client Relationship" OR DE "Physician Patient Relationship")
AND (DE “Decision Making“ OR DE “Informed Consent“ OR DE “Health Education“ OR DE “Participation“ OR DE “Individualized Programs“ OR DE “Decision Support Systems“ OR DE "Participative Decision Making")

CENTRAL thesaurus search (5 February 2020)

Adult
Immunization Vaccination
Communication Health Communication Persuasive Communication Interpersonal Relations Professional-Patient Relations Physician-Patient Relations Counselling
Decision Making, Shared Decision Making Clinical Decision Making, Decision Aids Decision Support Techniques Decision Support Systems, Clinical Informed Consent Choice Behavior Patient participation
Randomized Controlled Trial

PROSPERO search (5 February 2020)

(vaccin* OR unvaccine* OR immun* OR unimmun*) AND (Intervention OR Systematic Review):RT AND (Care_of_the_elderly OR education OR general_interest OR Health inequalities/health equity OR Mental health and behavioural conditions OR Public health including social determinants of health OR Service delivery OR Social care):HA

Clinicaltrials.gov search (5 February 2020)

Status: All Studies
Condition: vaccine OR vaccination OR immunization
Other terms: decision making OR communication

WHO search portal ICTRP search (5 February 2020)

vaccination AND communication (search 1)
immunization AND communication (search 2)
Resource Center http://www.nitag-resource.org/media-center/results?search=decision+making (5 February 2020)
Category: Systematic Review
Search: communication
